# Assessing the performance of local pharmaceutical systems: An analytical approach to improve access to medicine

**DOI:** 10.1177/27550834251371502

**Published:** 2025-09-27

**Authors:** Maarten Olivier Kok, Relmbuss Biljers Fanda, Rik Ubbo Lubbers, Margo van Gurp, Raffaella Ravinetto, Ari Probandari

**Affiliations:** 1 Erasmus School of Health Policy and Management, Erasmus University, Rotterdam, The Netherlands; 2 Amsterdam Public Health Research Institute, Amsterdam, The Netherlands; 3 Center for Health Policy and Management, Universitas Gadjah Mada, Yogyakarta, Indonesia; 4 Department of Health Sciences, University Medical Center Groningen, Groningen, The Netherlands; 5 Health, KIT Royal Tropical Institute, Amsterdam, The Netherlands; 6 Department of Public Health, Institute of Tropical Medicine, Antwerp, Belgium; 7 School of Public Health, University of the Western Cape, Cape Town, South Africa; 8 Department of Public Health, Faculty of Medicine, Universitas Sebelas Maret, Surakarta, Indonesia

**Keywords:** Pharmaceutical systems, pharmaceutical policy, decentralization, essential medicines, universal health coverage

## Abstract

Well-functioning pharmaceutical systems are crucial for ensuring universal access to medicines and their appropriate use. While existing frameworks for analysing pharmaceutical systems generally focus on the national level, in many countries, the core functions are often managed locally within a broader national framework. Despite this local focus, there has been no effort to conceptualize a ‘local pharmaceutical system’ as a distinct entity with its own goals, functions and operational components. A method for analysing and comparing the performance of local pharmaceutical systems (LOPHAS) within a country is still lacking. We aim to develop an analytical approach and framework to assess the performance of LOPHAS and guide improvements in access to essential medicines. We conducted an integrative literature review and consulted with purposively selected experts. We systematically searched for existing approaches for conceptualizing or assessing pharmaceutical systems and empirical studies in which these were applied and combined this with insights from 23 reviews and guidebooks suggested by experts to develop the LOPHAS approach and framework. We identified 13 existing frameworks and 16 studies that had applied these frameworks to analyse pharmaceutical systems. Building on these findings, we propose that a LOPHAS has six core functions: (1) local governance, (2) managing product supply, (3) financing, (4) developing human and physical resources, (5) appropriate dispensing and use of medicines and (6) monitoring performance. For each function, we defined operational components and indicators. The primary outcomes of a LOPHAS are access to medicine and appropriate use of medicine. The LOPHAS framework provides a practical tool for assessing and comparing the performance of LOPHAS. By identifying areas for improvement, it can guide policymakers, healthcare providers and local administrators in strengthening systems to ensure that essential medicines are accessible and used appropriately, supporting broader health goals.

## Plain Language Summary

Developing an Approach to Assess the Performance of Local Pharmaceutical Systems and Improve Access to Medicine

Why was the study done? Access to medicines is essential for public health, and well-functioning pharmaceutical systems play a critical role in ensuring that medicines are available, affordable and used appropriately. Most existing frameworks for assessing pharmaceutical systems focus on the national level, but many key functions, such as medicine distribution and use, are managed locally. Despite this, there has been no clear way to define or evaluate ‘local pharmaceutical systems’ as unique entities. This study aimed to fill this gap by developing a method to assess local pharmaceutical systems (LOPHAS) and identify ways to improve their performance. What did the researchers do? The researchers conducted a comprehensive review of the scientific literature to identify existing methods for analysing pharmaceutical systems and consulted experts to gather additional insights. Using this information, they developed an analytical framework, named the LOPHAS approach, that defines the key functions and indicators needed to evaluate LOPHAS. What did the researchers find? The researchers identified 13 existing frameworks and 16 studies that had applied these frameworks to analyse pharmaceutical systems. Building on these findings, they proposed six core functions of a LOPHAS: governance, supply management, financing, human and physical resource development, appropriate dispensing and use of medicines and performance monitoring. For each function, they outlined specific operational components and measurable indicators. How is this useful? The LOPHAS framework provides a practical tool for assessing and comparing the performance of LOPHAS within a country. By identifying areas for improvement, it can guide policymakers, healthcare providers and local administrators in strengthening systems to ensure that essential medicines are accessible and used effectively.

## Background

Around the world, countries struggle to ensure access to safe, quality-assured and affordable medicines for their populations. In many low- and middle-income countries, the average availability of medicines falls well below the 50% threshold in public facilities and below 70% in the private sector.^1,2^ Access to medicines also varies widely within countries.^3,4^ Studies consistently reveal significant disparities in medicine availability across districts, provinces and regions – differences that are often obscured by national averages.^4^–^6^ These local disparities exacerbate inequalities, as medication availability tends to be higher in affluent urban areas and lower in rural and remote areas, where residents typically have lower incomes and less access to health services.^7^–^10^ Limited access to medicines and high prices pose serious risks to patients’ health by increasing the likelihood of non-treatment, inadequate treatment and catastrophic health expenditure. Moreover, poor availability in public facilities and licenced pharmacies drives patients to seek medications from unregulated outlets, heightening the risk of encountering costly, expired and substandard or falsified medications.^11,12^

Ensuring access to quality-assured and affordable medicines and promoting their appropriate use requires a well-functioning pharmaceutical system.^13^ Drawing upon work by Hafner et al.,^14^ we define a pharmaceutical system as the people, practices, structures and resources and their interactions within the broader health system that aim to ensure equitable and timely access to safe, effective, affordable and quality-assured pharmaceutical products and related services that promote their appropriate use to improve health outcomes. While it can be useful to describe a pharmaceutical system as a set of components, the mutual dependencies and interconnected practices and structures together make it function as a system.^14^–^16^ Even when pharmaceutical systems are not deliberately designed or centrally organized, they do emerge as de facto systems through the patterns of actions and interactions of those who procure, store, distribute, prescribe and use medicine within the larger world in which they are embedded.^17^ Like most systems involving humans, pharmaceutical systems are complex, contingent and locally specific and co-evolve with the larger societal structures in which they operate, such as the health system, pharmaceutical market and political economy.^15,18^ Several pharmaceutical system frameworks have been developed,^14,19^–^22^ and empirical studies have demonstrated their utility in guiding analyses of national pharmaceutical systems.^23^–^26^

While the national level may seem the logical focus from a policy perspective, many countries have decentralized the organization of core functions of their pharmaceutical system that directly affect access to medicines to a more local level. For instance, in Indonesia, the local authorities in each of the 514 districts are responsible for forecasting local medicine needs, procurement and distribution.^7,27^ These districts operate relatively autonomously as local systems, with large variations in terms of medicine availability, affordability and accessibility.^28,29^ Comparable decentralized systems are in place in several other, often larger, countries like Brazil, India, the Philippines and Nigeria.^30^–^32^ In this article, we present a strategy for assessing the performance of local pharmaceutical systems (LOPHAS) and exploring the underlying causes of disparities in access to medicine across different regions.

Core functions of a pharmaceutical system that are typically organized at the local level include forecasting medicine needs, procuring, storing and distributing medicines to facilities.^5^ Local organization of these functions offers notable advantages, allowing for tailored medicine supply to meet local needs and circumstances and accountability to local communities.^33,34^

Even in countries with decentralized systems, certain functions remain centralized at the national level, such as regulating market authorization, vigilance, trade and taxation of pharmaceutical products.^14^ Furthermore, establishing rules and regulations regarding medicine pricing, distribution and prescription practices is commonly managed at the national level.^5^ Although the national level holds formal authority, local authorities and stakeholders may still wield significant influence over these functions, as they are often tasked with interpreting and enforcing policies and regulations within their local contexts.^31,35^ There are also system functions organized at both the national and local levels, such as the financing of publicly procured medicines, which is often sourced from national and local budgets.^25,36,37^

In addition to the local organization of system functions, local circumstances can significantly influence the supply, pricing and accessibility of medicines.^38,39^ Challenges like poor infrastructure, distance to suppliers and local insecurity can complicate and escalate the cost of medicine supply in both the public and private sectors.^4,31,40^–^42^ Considering these local conditions is essential for understanding the disparities in access to medicines across different regions within a country.

While many core functions of pharmaceutical systems primarily operate at the local level, there is presently, to the best of our knowledge, no dedicated analytical framework designed to evaluate the performance of a LOPHAS. Such an approach and framework have the potential to offer a standardized method for mapping and comparing the performance of local subsystems within the same country, facilitating learning from variations in how local systems operate, perform and develop over time. In addition, it may offer guidance when designing and testing interventions to enhance pharmaceutical system performance.

The aim of our study is to develop an analytical approach and framework for measuring, analysing and comparing the performance of LOPHAS. To achieve this, we conducted an integrative review, synthesizing pharmaceutical system frameworks, empirical studies and insights from purposively selected experts.^43^ Drawing upon these insights, we present a LOPHAS as a mixed, nested, complex adaptive system (CAS), that should be analysed using a holistic perspective, taking into account the dynamic relationships between medicines, other components of the health system and markets at multiple levels. To guide analyses and comparison of LOPHAS, we defined the functions and operationalized components of LOPHAS, which we then linked with relevant indicators. As an analytical strategy, we propose assessing disparities among local systems within a country, integrating performance metrics with data about how local systems are organized and supplementing quantitative evaluations with qualitative investigations into actual practices and interactions among system components and levels.

## Methods

We developed the LOPHAS approach and framework in three stages. We started with conducting an integrative review to identify existing system approaches and frameworks that conceptualize and describe a pharmaceutical system. We chose to conduct an integrative review since it allows for the inclusion and integration of insights from a variety of both theoretical and empirical literature to understand a phenomenon of concern more fully.^43^ Next, we searched for empirical studies in which these frameworks are used, and empirical analyses that explicitly use a systems approach to analyse the functioning of a pharmaceutical system or supply chain. In the second stage, we read, analysed and compared the identified system frameworks, assessed the main domains and functions described in each framework and summarized the insights from the empirical studies that were relevant to the operation and performance of LOPHAS. Using the constant comparative method of analysis, we synthesized these insights into a first draft of an analytical framework that describes the main functions of a LOPHAS. We continued by formulating indicators for each of these core functions. In the third stage, we shared the description of the LOPHAS framework and its functions and indicators with seven purposively selected experts and asked them to provide feedback and suggestions for improvement. In section ‘Results’, we describe the framework and the core functions, which result from the integrative and interpretive development process.

### Literature search

The search strategy is depicted in Table 1 with search terms and databases. Searches were conducted in PubMed, SCOPUS and Google Scholar. Our study had the following inclusion and exclusion criteria:

- Explicitly using a systems perspective.
- English literature.
- 1990 until the end of June 2024.
- Peer-reviewed research articles.
- Grey literature, which is explicitly based on empirical research.

**Table 1.** Search terms and databases.

Databases	Search terms			
PubMed	Pharmaceutical systems			
Google Scholar	Access to medicine(s)			
	Access to pharmaceuticals			
	Framework			
	Strengthening			
	Subnational			
	District			
	State			
	Province			
	Decentralized			
	Local			
Search strategy				
Subject		Concept		Context
Pharmaceutical systemsORAccess to medicine(s)ORAccess to pharmaceutical(s)	AND	Framework	AND	Subnational
		OR		OR
		Strengthening		District
				OR
				State
				OR
				Province
				OR
				Decentralized
				OR
				Local

An iterative process was employed, using the results and bibliographies of relevant studies to guide subsequent searches. The search was considered saturated when no noticeably new publications emerged. The snowball method was applied by consulting the bibliographies of key studies and identifying studies that cited them to uncover additional relevant research.

Relevant studies were independently searched by three authors, and the titles and abstracts were screened for relevance. Studies that met the criteria were compiled into a CSV file in Excel. Studies were included if they assessed the functioning or strengthening of a pharmaceutical system using a system approach. Once search saturation was reached, the CSV file was screened for duplicates, which were then removed. The relevant studies were organized in Zotero^44^ and reviewed by two authors. Articles that met our inclusion criteria were included in the final review and analysis.

### Data evaluation

Since an integrative review allows for the inclusion of many different types of research, we used the Mixed Methods Appraisal Tool (MMAT) to appraise the quality of studies.^45,46^ Developed to evaluate qualitative, quantitative and mixed methods research, the MMAT is specifically designed for reviews that include diverse study designs. It enables the appraisal of five categories of studies: qualitative research, randomized controlled trials, non-randomized studies, quantitative descriptive studies and mixed methods research. Each category includes five specific methodological quality criteria. In our study, we used the 2018 MMAT version. Most of the included studies had used a qualitative approach, for which there are five MMAT criteria: (1) assess whether the approach is appropriate, (2) data collection is adequate, (3) findings are grounded in data, (4) interpretations are supported by evidence and (5) all elements are methodologically coherent. Two authors independently appraised the selected articles, then compared their assessments and resolved any discrepancies through discussion to reach consensus. Articles deemed of insufficient quality by both reviewers were excluded from further data analysis.

### Data analysis

The following information was extracted from all included studies: PMID/DOI, title, authors, year of publication and the journal. In addition, frameworks and their descriptions were extracted, along with any data related to the functioning of pharmaceutical systems at the local level. The critical components, functions and goals of these frameworks were analysed and compiled into a table (Supplementary File 1). The empirical studies were thoroughly reviewed to highlight important insights on the functioning of LOPHAS, which were also extracted. Three authors conducted the data analysis and developed the framework.

#### Expert consultation and validation

In the third stage, we shared the initial LOPHAS approach and framework with seven purposively selected experts. The expert consultation was designed to provide an additional layer of critical reflection and empirically grounded feedback to strengthen the development of the LOPHAS approach and framework. We set out to select experts who had extensive practical experience in strengthening pharmaceutical systems at both national and local levels across a variety of country contexts. Diversity in geographic and institutional background was a key consideration in our selection process. We followed a purposive sampling approach and continued inviting new experts until we reached saturation and their contributions confirmed the robustness of the approach, framework and functions. The consulted experts have contributed to system-strengthening efforts across all continents and have held relevant senior positions in prominent national and international organizations. We asked these experts to carefully assess the approach and framework and provide feedback and suggestions for improvement.

A key suggestion of these experts was not only to include publications that explicitly used a systems approach but also to draw upon the broader literature on access to medicine. Based upon the expert consultation, we identified 23 review articles and two handbooks^5,47^ that provide further empirical insights into improving access to medicines. After gathering feedback from all experts and carefully reviewing the articles and handbooks, we compared and triangulated the core elements of the LOPHAS approach. We discussed differences and suggestions until a consensus was reached and the approach and framework were finalized.

## Results

### Included publications

**Figure 1.**
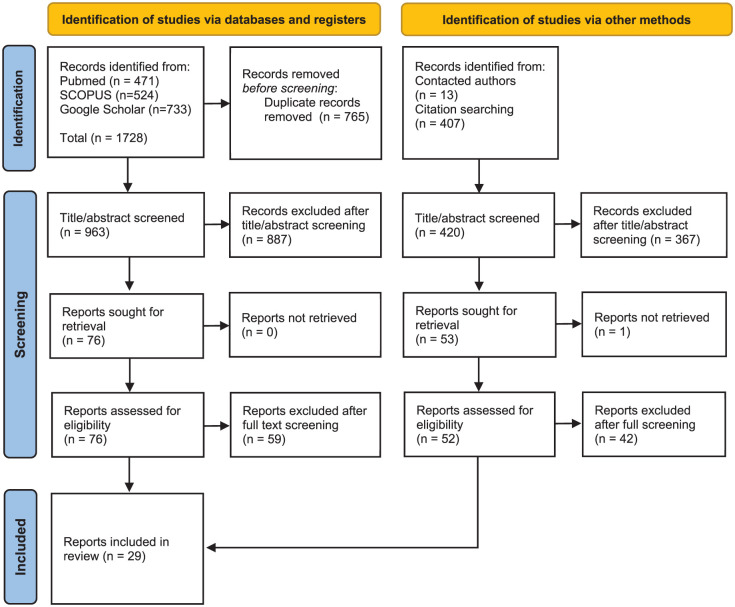
PRISMA flow chart of included studies.

Our systematic search strategy resulted in 29 studies for our integrative review (Figure 1). In total, 13 studies provide some sort of framework to analyse the functioning of a pharmaceutical system (Table 2). A detailed comparative analysis of the critical components, functions and goals of a pharmaceutical system is available in Appendix 1. We identified 17 empirical studies that explicitly used a systems perspective (Supplementary File 2). There were three types of studies: analyses of a pharmaceutical system’s situation at a specific point in time, studies that evaluate barriers and enablers to accessing medicines and studies that assess pharmaceutical system interventions using a system approach. We found two studies that provided a novel framework and empirical data. None of the studies specifically aimed to describe local functions within a national system. The consultation with experts resulted in an additional 23 reviews and two handbooks, which were used to develop, enrich and triangulate our approach further. The 13 frameworks, empirical studies and reviews provide a rich mosaic of insights, based upon concrete experiences in dozens of countries, on all continents, relevant to analysing pharmaceutical systems. Some large middle-income countries, which decentralized pharmaceutical systems, are represented in several empirical studies, including Indonesia, Brazil and Nigeria.

**Table 2.** Existing pharmaceutical system frameworks/assessment tools.

Framework	Aim(s)	Method to develop framework
Rapid pharmaceutical management assessment: an indicator-based approach (Management Science for Health, 1995)^48^	To present an indicator-based approach for rapidly assessing pharmaceutical management systems and programmes	1. Recommendations from panel 2. Filter 200 indicators to 33 3. Expert consultations. 4. Conduct a field test
Improving access to essential medicines – a framework for collective action (WHO, 2004)^49^	To guide and coordinate collective action on access to essential medicines	No information
Functional components of a pharmaceutical system framework (Edgar Barillas, 2005)^50^	To ensure the effective and efficient functioning of the pharmaceutical system within the context of health sector reform	No information
Using indicators to measure country pharmaceutical situations (WHO, 2006)^21^	To summarize and provide a picture of the situations of different pharmaceutical sector components and the current status of national drug policies	Tested in 2003 and 2006 in 146 countries
The regional framework for action on access to essential medicines in the Western Pacific 2010–2015 (WHO, 2009)^51^	Multiple, including responding to country needs and challenges in the development and implementation of activities, based on expressed principles and grouped under the following components: policy and access to essential medicines, quality assurance and rational selection and use	Expert consultations and in-depth reviews
Pharmaceutical supply management framework (Management Science for Health, 2012)^52^	The aim of the framework is to assess the functioning of the supply system of a pharmaceutical system	Unclear
ATM from a health system perspective: a conceptual framework (Bigdeli et al., 2013)^15^	The aim is to embed ATM in the wider health system-strengthening debate, as a system approach to improving ATM seeks to ensure that policies are more effective and generate longer-term equitable and sustainable results	Literature review
SIAPS PSS framework (SIAPS, 2013)^53^	To depict a comprehensive set of dynamic relationships among five health system building blocks, with a medical product building block overlay representing SIAPS’ technical focus on strengthening pharmaceutical systems and services within a health systems context, aligned with partner country strategic plans and USG/USAID health-related goals	Unclear
Lancet commission progress indicators (Wirtz et al., 2017)^3^	To present indicators to assess the progress on essential medicine policies	An expert commission
PSS measurement framework (SIAPS, 2018)^54^	To have created a measurement framework and corresponding indicators for determining whether investments in pharmaceutical system strengthening are contributing to the development of stronger, more sustainable pharmaceutical systems	Literature review and expert consultations
Action-oriented pharmaceutical sector strengthening cycle (Oteba et al 2018)^55^	To depict prioritized action areas, levels and goals of a pharmaceutical system to strengthen them	Unclear (based on a USAID framework)
Access to medicines framework (Afzali et al., 2019)^20^	The aim of this study was to collect and compile applicable indicators and impart a comprehensive framework for assessing access to medicine	Literature review, focus groups and a Delphi study
Analytical framework for qualitative evaluation of access to medicines from a health system perspective (Joosse 2024)^56^	To develop a framework specifically designed to identify barriers, facilitators and drivers to access to medicines from a country’s health system perspective	Not specified. Presented as a by-product of a study on the barriers and facilitators to medicines in South Africa

### Three types of pharmaceutical system perspectives

Careful analysis of the 13 system frameworks shows that they are grounded in three different system perspectives, which can be referred to as (1) mechanistic, (2) teleological and (3) CAS perspective. Before laying out our analytical approach and the LOPHAS framework, we first characterize these three types of system perspectives.

The first type of framework to analyse a pharmaceutical system is rooted in a mechanistic systems perspective. These mechanistic frameworks emerged from the basic idea that enhancing access to medicines necessitates the consideration of diverse actors and factors operating in concert as a system.^13^ In these frameworks, analysts outline these components and their functions within the system.^57^ A mechanistic perspective views a system as a relatively static entity consisting of several parts with defined functions and structures. The empirical studies that use this perspective tend to break down the pharmaceutical system into smaller, more manageable parts. Cause-and-effect relationships are often perceived in a linear manner, assuming that changes in one part of the system directly lead to direct effects in other parts.

A second type of pharmaceutical system approach starts from a teleological perspective. An early example is the Rapid Pharmaceutical Management Assessment approach, published in 1995.^48^ A teleological perspective refers to a way of understanding systems by focusing on their purpose, goals and outcomes. Pharmaceutical systems are viewed as purposeful entities that are designed to achieve specific objectives, such as providing access to medicines.^54^ Teleological systems analysis emphasizes the goals or desired outcomes that a system is designed to achieve. Systems are seen as exhibiting purposeful behaviour directed towards achieving their goals. A teleological approach assumes that understanding the goals and processes of a system is essential for effective decision-making and intervention. These teleological perspectives assume that pharmaceutical systems may evolve over time as a result of external influences or deliberate interventions to strengthen system performance.

While teleological approaches can be valuable for analysing a pharmaceutical system, they are not without criticism. One critique is that the emphasis on a predetermined goal or purpose overlooks the ambiguity of goals and the fact that goals may vary among different actors in the system. A second issue is that analyses that use a teleological perspective tend to overlook the emergent properties and behaviours that arise through the interactions of system components. This is illustrated clearly by Bigdeli and colleagues, who show how this can lead to the neglect of unintended consequences, interdependencies and contextual factors that can have a major influence on access to medicine.^15^ A teleological perspective thus risks oversimplifying the complexity of pharmaceutical systems by reducing them to an intended purpose, overlooking the intricacies of their structure, dynamics and interactions. An additional criticism is that, as goals are unclear and diverse and feedback loops occur, a teleological perspective may struggle to provide accurate predictions about the behaviour of a system. While teleological systems perspectives can be useful, it is important to recognize their limitations and consider alternative perspectives that complement their strengths.

A third perspective on pharmaceutical systems draws from CAS thinking. Bigdeli and colleagues provide an example of a CAS perspective in their study of how interactions between different health system components influence access to medicine.^15^ Complexity theory acknowledges that social systems exhibit emergent properties, non-linear dynamics and self-organization. Instead of assuming that pharmaceutical systems are deliberately designed and exist to achieve a predetermined goal, a CAS perspective focuses on the de facto systems that have emerged in practice through the patterns of actions and interactions of those who procure, distribute, prescribe and use medicine. Following a CAS perspective, a pharmaceutical system comprises numerous interacting and mutually dependent components that adapt and evolve in response to changes in internal dynamics and the environment. Such systems are shaped by non-linear relationships, feedback loops and unintended consequences, which cannot be fully predicted or explained. A CAS perspective encourages analysts to study the actions of actors, the diverse aims and mechanisms that shape their behaviour, feedback loops and the role of context in shaping system dynamics. It promotes examining how a system performs in practice and how it is influenced by larger socioeconomic, political and environmental contexts.

Our approach assumes that each of these three system perspectives – teleological, mechanistic and CAS – provides valuable insights for analysing and strengthening LOPHAS. While we primarily focus on the system’s purpose and outcomes (teleological perspective), we also describe the system’s components (mechanistic perspective) and explore their relationships. In addition, we conduct in-depth analyses of system properties, dynamics and how systems emerge and evolve (CAS perspective).

### Conceptualizing and defining a LOPHAS

A LOPHAS can be viewed as a subsystem nested within a multilevel structure of larger, complex and evolving systems, such as the health system and the national and international pharmaceutical markets and policies. These systems operate within a broader, national and international sociopolitical context. As a subset of these systems, a LOPHAS includes all structures, people, resources and processes at the local level that contribute to ensuring access to pharmaceutical products and related services. LOPHAS emerges when the core functions of the pharmaceutical system are organized and managed at the local level.

Building on this description and existing research, we define a LOPHAS as the people, structures, resources, policies and interactions that ensure local access to medicines, their appropriate use and health-related services to improve health outcomes.

**Figure 2.**
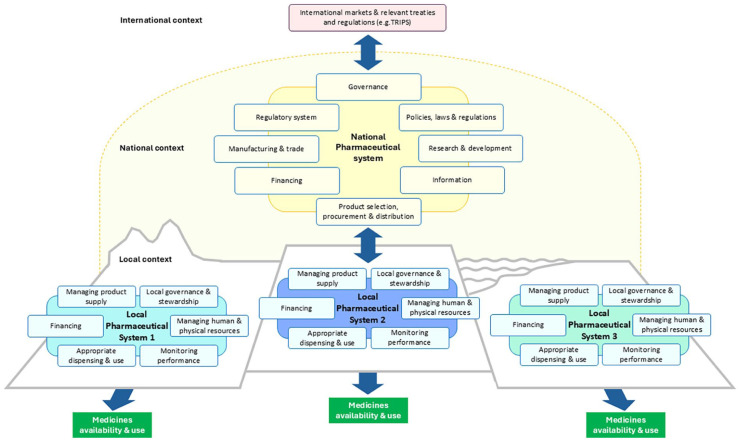
The LOPHAS framework.

Our analytical framework of LOPHAS is synthesized in Figure 2. The figure tries to illustrate LOPHAS as one of many subsystems embedded within the national system, as well as place the LOPHAS into the broader health system and societal context.

### A four-step approach to assessing the performance of LOPHAS

To leverage the strengths of each system perspective and generate valuable insights, we propose a four-step approach for analysing LOPHAS.

#### Step 1: Mapping stakeholders and describing the formal organization of the system

The first step starts with mapping stakeholders and describing the formal organization of a pharmaceutical system. The purpose of the stakeholder mapping is to identify the entities involved in or impacted by the LOPHAS. In general, one can identify the core stakeholders, supporting stakeholders and end users.^58,59^ The core stakeholders are all entities directly involved in the pharmaceutical supply chain, such as government agencies, manufacturers, wholesalers, retailers, pharmacies and healthcare providers. The supporting stakeholders are the organizations that indirectly impact the system, such as non-governmental organizations (NGOs), donor agencies and community leaders. The end users are the patients and communities who access and use the medicines and related services.

There are significant differences between pharmaceutical systems in different countries. This first step includes identifying which functions are organized at the national level and which are managed locally and clarifying the distribution of roles and responsibilities. To gain insight into a pharmaceutical system, it is also useful to map where medicines are produced (domestic or elsewhere), how they are regulated, how supply chains operate and are overseen, how medicines reach patients, how the system is financed, whether there is a health insurance system and what role the public and private (either for-profit or not-for-profit) sectors play in this. In some countries, it may also be relevant and possible to describe the informal (unregulated) market.

For this first step, the World Health Organization (WHO)^13^ and Systems for Improved Access to Pharmaceuticals Services (SIAPS) PSS^54^ systems can be used as inspiration, which provide a comprehensive overview of the core components and functions of a national pharmaceutical system.

In this initial stage, it is beneficial to clarify the specific aims of the system analysis (e.g. learning, improvement, accountability) and identify the intended users of the analysis. Engaging these intended users in contextualizing the analytical approach and designing the next steps can ensure the analysis is relevant and increases the likelihood the findings are actionable.^60,61^

#### Step 2: Empirical analysis of the performance of LOPHAS

The second step is an empirical analysis of how local systems operate and the extent to which intended goals, such as medicine availability and access, are achieved. For this second step, we developed the LOPHAS framework, which includes a detailed description of the functions and goals of the pharmaceutical system at the local level (see Table 3).

**Table 3.** Functions and operational components of a local pharmaceutical system.

Functions	Operational Components
Local governance and stewardship	- Develop a strategic plan and priority actions to foster ATM - Coordinate with other governmental actors and levels to foster ATM - Map, engage and coordinate with non-governmental and private actors to foster ATM - Contextualize and implement national policies and regulations - Establish local mechanisms for transparency, accountability and responsiveness
Managing pharmaceutical product supply	- Select which products are needed in which health facilities - Establish efficient planning systems and forecast product needs - Procure pharmaceutical products - Store pharmaceutical products and monitor stock and quality - Distribute pharmaceutical products to dispensing units - Track prices and supplier performance
Financing	- Coordinate and secure sustainable funding for the procurement and contracting of pharmaceutical products, resources and services - Allocate and distribute expenditures to pay for pharmaceutical products and related resources and services - Monitor revenue, expenditures and prices and cost-effectiveness of investments in pharmaceutical products, resources and services
Developing and sustaining human and physical resources	- Develop, manage and support a competent pharmaceutical workforce - Develop, manage and sustain the physical and technological resources needed for pharmaceutical operations
Ensuring appropriate dispensing and use	- Promote appropriate prescribing, dispensing and retail practices and service delivery that supports appropriate use - Contribute to monitoring of practices and quality in the regulated supply chain - Contribute to inspection of retail, marketing and dispensing practices in unregulated outlets and the enforcement of regulations
Monitoring performance	- Monitor appropriate prescribing, dispensing and retail practices and service delivery that supports appropriate use - Monitor availability of pharmaceutical products in dispensing units - Identify and evaluate problems and solutions to improve access to and use of medicine - Evaluate and monitor ethical standards for pharmaceutical system and access to medicine - Collect and interpret other data relevant for decision-making

The second step involves analysing the relationship between the functioning of local systems and the various dimensions of access to medicine, including availability, accessibility, affordability and acceptability. Later, in this article, we describe the goals and core functions of LOPHAS in detail, along with several operational components and indicators for each function. These operational components and indicators are designed as suggestions that can be adjusted for specific contexts. Together with stakeholders, an analyst needs to select the specific metrics for evaluating the effectiveness, efficiency, accessibility and quality of the LOPHAS. This could include indicators based on health outcomes, medicine availability, service quality, patient satisfaction and affordability indicators. Depending on the situation and the purpose of the analysis, analysts need to consider the broader (political, socioeconomical, cultural, infrastructural and geographical local context), which can influence the functioning of local systems, the specific challenges they face and the most relevant dimensions of access to medicines.

#### Step 3: In-depth qualitative analysis

The third step involves studying the actual practices within the pharmaceutical system. In many countries, there is a gap between how a system is supposed to function on paper and what actually happens. Financial incentives, complex supply chains, unclear regulations, poor infrastructure, failing technology, local insecurity, lack of (skilled) human resources, incompetence and corruption can all lead to medicines being unavailable and patients spending significant amounts on expensive alternatives in private and unregulated outlets.^3^

In-depth qualitative analyses are necessary to gain insights into actual practices and understand how the system functions.^15,56^ Through interviews and observations, researchers can discern what actors do, how incentives shape their practices, the feedback loops and unexpected outcomes that arise, how various layers and functions within the health system interact and how broader structures and dynamics influence these within the context.

#### Step 4: Integrating insights and developing actionable steps for improvement

In the final stage, analysts can integrate the insights from previous steps to understand how local systems perform and design actionable steps for improvement. The exact focus of this fourth stage depends on the specific goals of the system analysis, such as learning from comparisons, tracing the development of local systems over time or informing interventions for system strengthening. Engaging intended users and other stakeholders can help validate, interpret and contextualize findings, leading to the development of relevant and actionable insights.

These insights can inform the design of targeted interventions, such as optimizing supply chain processes, expanding access points or training staff. The data collected in Step 2 can serve as a benchmark and a foundation for a monitoring system that tracks improvement efforts and supports necessary adaptations. This monitoring should be locally tailored, using progress dashboards and feedback loops with frontline healthcare workers and patients to drive better health outcomes.

##### Key outcomes of a LOPHAS

The goal of the LOPHAS is to ensure access to medicines and appropriate use of medicine. *Access to medicine* within the health sector is most commonly defined as geographical and financial accessibility, availability and cultural acceptability of quality services and products.^22^ The selection, number and formulations of medicines included depend on each country’s national Essential Medicines List (EML) and its policies regarding availability in specific healthcare facilities.^62^ Access to pharmaceutical products is commonly measured by the following indicators, which measure key domains of access (another domain is quality, which is not commonly assessed at the local level):^3,20,21^

**Table:** 

• Median availability of a basket of essential medicines (%). • Distance to nearest medicine dispensary (in km, depending on the specific medicine). • Community perception on access to health care. • Median consumer price ratio of a basket of essential medicines.

*Use of medicine* is described as prescribing, dispensing, or selling and consumption or end-use by the patient.^14^ In line with previous research, we include the fact that the use of medicine should be appropriate and cost-effective. Appropriate since medicine can be prescribed or dispensed inappropriately (wrong substance, dosage, administration route, time period, etc.) and/or consumed inappropriately (e.g. wrong self-prescription and incomplete treatment cycle), as is a common occurrence.^5,15^ Cost-effective since, in many countries and/or by specific product, the affordability of medicine is low. A high proportion of dispensed generic medicine indicates better affordability. Based upon the integrative review and expert consultations, we suggest four general indicators for this outcome measure:^20,21^

**Table:** 

• Number of prescribed essential medicines dispensed per 1.000 pop. • % Medicines prescribed as generics. • % Medicines prescribed from Essential Medicine List. • % (exact or estimate) Population with unmet medicine needs.

### Key functions of a LOPHAS

Our analytical framework outlines six essential functions for a LOPHAS: (1) local governance and stewardship, (2) managing pharmaceutical product supply, (3) financing, (4) developing and sustaining human and physical resources, (5) ensuring quality and promoting appropriate use and (6) monitoring performance. Table 3 provides an overview of each function, including its operational components.

#### Local governance and stewardship

The governance function focuses on the coordination, steering and oversight provided by local health authorities who are tasked with ensuring that the pharmaceutical system operates efficiently, effectively and transparently and aligns with national and local health objectives. At the local level, a district or provincial health office (or equivalent function) typically shoulders the responsibility for overseeing the performance of the local system.^21,47^ In most countries, some policy guidance is typically derived from the national level.^25,55,63^ The local authorities are tasked with crafting a vision and a strategic plan tailored to the local context, coordinating with stakeholders across various levels and crafting policies concerning the selection, management, procurement and distribution of pharmaceutical products for public facilities. A well-defined vision guides activities towards the overarching goal of the system: ensuring access to essential medicines and fostering their appropriate use to enhance health outcomes. In line with its priorities, local authorities have to formulate concrete plans to achieve results. Developing these plans requires a thorough understanding of the local situation, health needs, opportunities, obstacles and their root causes that need to be overcome, as well as awareness of the sphere of influence of the authorities in charge.^5^

A key task is coordinating with other national and local actors to foster collaboration and collectively improve pharmaceutical services. Decision-making responsibilities between national and local authorities can vary. Coordination is essential, as local authorities play a vital role in implementing national policies and regulations while providing data and input for national decision-making.

Coordination is also needed with other public and private actors within the local system. This involves scanning and mapping the pharmaceutical landscape – including public, private and unregulated/informal entities – and having insight into which products and services are provided by whom.^47^ Effective engagement with the private sector is needed to achieve public health goals, such as appropriate dispensing of antibiotics. Building relationships with the private sector involves being a reliable partner, fostering genuine engagement and transparent decision-making and communicating clearly about priorities, regulations and guidelines.^64,65^ A final component of the local governance of pharmaceutical systems is establishing mechanisms for transparency, accountability and responsiveness in pharmaceutical operations, financial transactions and the effective use of resources in coordination with national mechanisms and regulations.

The following indicators could be useful to assess local governance and stewardship:

**Table:** 

• Mapping the concerned local stakeholders and their respective roles in the local system. • Local health authorities have developed a vision and a strategic plan for achieving ATM and appropriate use. • Local health authorities have formulated context-specific plans to achieve this vision. • Progress reports on local priorities and policy plans are publicly published. • Local health authorities coordinate with relevant governmental and non-governmental actors at local and national levels to achieve ATM and appropriate use. • Local health authorities have established explicit mechanisms for transparency and accountability about pharmaceutical operations, financial transactions and use of resources.

#### Managing pharmaceutical product supply

A core function of LOPHAS is to manage the supply of pharmaceutical products. This involves systematically predicting and tracking the demand for pharmaceutical products and selecting, procuring, storing and distributing these products to dispensing units, along with related services. This core function is interconnected with all other functions and directly contributes to the goals of LOPHAS – ensuring access to medicine and promoting the appropriate use of medicine.^48,54^

The initial selection of pharmaceutical products occurs at the national level, where authorities establish an EML. In many countries, this process is informed by the WHO Essential Medicines Model List.^62^ Local authorities tend to be responsible for selecting which products and formulation of the EML are prioritized in the local context and which products will be available in which facilities. Following the selection of pharmaceutical products, local authorities play a key role in forecasting, procuring, storing and distributing products to dispensing units. Any issues in the supply chain, whether due to inaccurate forecasting, incorrect procurement, inadequate storage capacity or insufficient distribution, can result in medicine shortages. The operationalized components of procurement, storage and distribution are underpinned by various supporting functions, including the availability of adequate human, physical and financial resources, effective local governance and stewardship, quality assurance and continuous monitoring and, if necessary, targeted actions to strengthen the performance of the system.^14^

The operationalized components of forecasting and monitoring local pharmaceutical product needs are critical and unique functions of LOPHAS. In some countries, local health authorities forecast medication needs (for the public sector) for a given period, typically 1 year, which are then compiled by national authorities responsible for providing the required products to local authorities.^66^ In other countries, medicines for public facilities are procured locally, or there is a mix of central and local procurement. Poor forecasting can result in stock-outs or oversupply and expired medicines. Estimating the local medication needs depends on up-to-date data on medicine use, stocks and disease incidence and prevalence^5,24^ and accurate knowledge of the contribution of other stakeholders, such as NGOs or other implementers. Monitoring medication stocks can help to refer patients to facilities with adequate stocks, thus improving access.^24,27^

Indicators are essential for measuring the components of forecasting, procurement, storage and distribution of medicines to assess the pharmaceutical product supply.

The following indicators could be useful to assess the local management of product supply:

**Table:** 

• Mapping of different actors involved in supply and procurement across sectors. • Local authorities list which medicines are essential and should be available in which facility. • Reliable local data on medicine needs and use exists. • Reliable local data on medicine stocks exists. • System in place to align procurement with up-to-date disease prevalence and incidence. • Organization to forecast has adequately trained human resources to forecast medicine. • % of medicines procured from EML. • % of medicines procured that are registered or authorized with the NRAs (this should be 100%). • Adequate facilities to store medicine. • Organization tracks medicine prices and monitors supplier performance. • % of medication expired, damaged or stolen of a basket of essential medicines. • Mean % availability of a basket of essential medicines.

#### Financing: securing and allocating funds

The financing function involves managing resources to ensure adequate and sustainable funding for purchasing pharmaceutical products, covering related services, supporting human resources and meeting other costs essential to the LOPHAS.^36,67,68^

Financing for pharmaceutical products in public facilities typically originates from various channels. Often, national governments allocate funds to local authorities, which then combine these with local resources to procure medicines.^23^ In addition, there are specific medications that are procured nationally and distributed to public facilities. Besides the public system, most countries also have a vibrant private market, where private healthcare providers, social enterprises, pharmacies and other medicine outlets acquire medicines.^5,69,70^ Financing in this sector usually stems directly from patients and health insurers and sometimes from other sources such as charities and donors.^5^

A core component of this function is coordinating and securing funding for procuring and contracting pharmaceutical products, resources and services. The authorities in charge need to pool, allocate and distribute resources, purchase pharmaceutical products and pay for related services and resources.^71,72^ Transparent monitoring of revenue, costs and prices is crucial to controlling spending, supporting decision-making and promoting accountability in financial transactions and resource use.

The following indicators could be useful for assessing the local financing function:

**Table:** 

• Mapping of the concerned local stakeholders and financing flows. • Medicine expenditure per person by public sector. • Back-up funding from national or local government is available. • Out-of-pocket expenditure on pharmaceuticals as a percentage of total pharmaceutical expenditure. • % of people enrolled in insurance scheme. • System in place to monitor revenue, expenditures and prices across sectors. • Adequacy and transparency of mark-ups.

#### Developing and sustaining human and physical resources

Developing, supporting and sustaining a competent pharmaceutical workforce is essential for a well-functioning pharmaceutical system at all levels. A skilled pharmaceutical workforce is critical for the selection, procurement and management of medicine and for providing pharmaceutical services and promoting the appropriate use of medicine.^55^

Normally, policies and regulations for training new health professionals, including pharmacists, pharmacy technicians or pharmacy assistants, as well as administrators in health systems, are set at the national level. National governing bodies are tasked with accreditation of training programmes and ensuring that training is provided in accordance with accepted norms, guidelines, standards and regulations.^20^ In some countries, local governments also invest in training new staff by (co)funding and supporting local universities and training programmes. Additional training is often delivered through short courses, in-service programmes, remote training and continuous education to enhance pharmaceutical skills and competencies.^73,74^ However, in many countries, companies attempt to influence medicine provision through such training. Local authorities must ensure that healthcare professional training prioritizes evidence-based prescription criteria and, ultimately, patient health.

Local healthcare authorities play a vital role in managing the pharmaceutical workforce by recruiting, hiring, deploying and supporting pharmacy professionals, as well as monitoring and evaluating their performance. Insight into the ratio of pharmacy professionals per inhabitant offers a valuable measure to evaluate the human resource capacity of the LOPHAS. These data can inform strategies for the development and distribution of pharmacy professionals.

The following indicators could be useful to assess the local development of human resources:

**Table:** 

• Number of pharmacists per 10.000 pop. • Number of pharmacy practitioners per 10.000 pop. • Number of pharmaceutical technicians/assistants per 10.000 pop. • % of dispensing facilities in which a pharmacist is present. • The relative distribution of pharmacist – considering aspects such as public or private sector and rural and urban areas.

Physical resources are vital for pharmaceutical operations, including the adequate storage, distribution and proper dispensing of medicines, which are essential for ensuring access to medicine. Inadequate access to dispensing facilities can force people to travel long distances, resulting in the loss of time and money to obtain medication. Authorities overseeing LOPHAS are responsible for ensuring an adequate number of licenced pharmacies by developing, supporting and maintaining public pharmacies, as well as licencing private pharmacies and other outlets. In remote and underserved areas, unlicensed dispensing units, such as informal drug vendors and midwives, also provide medicines.^12^ Local authorities need to determine if such outlets truly fill a gap and can try to engage them through training, upgrading and licencing constructively.

The following indicators could be useful to assess the physical resources involved in pharmaceutical operations:

**Table:** 

• Number of pharmacies per 10.000 pop. (specified for public and private). • Number of licenced pharmacies per 10.000 pop. • Number of unlicensed dispensing units per 10.000 pop. • Distance to nearest pharmacy (from communities). • Distance to nearest dispensing unit (from communities).

#### Ensuring appropriate dispensing and use

Local health authorities need to ensure the adequate use of pharmaceutical products and services, which includes promoting adequate storage and distribution practices and appropriate use of medicines.

Ensuring the quality of medicines is primarily the responsibility of national regulatory authorities, which establish and enforce safety, efficacy and quality standards.^26,55^ Manufacturers, distributors and pharmacies must comply with these standards, regulations and guidelines across manufacturing, distribution and pharmaceutical practices.^75^ Unregistered, expired and substandard or falsified products may become more prevalent in countries with limited regulatory enforcement and unmet demand.^76^–^78^ Local health authorities can support regulators by reporting suspected or expired products, assisting with inspections, regulatory enforcement and monitoring prescription, marketing and retail practices.^73^

The following indicators could be useful to assess local quality assurance:

**Table:** 

• Regular inspection of good storage and distribution practices. • Regular reporting of expired, unlicensed and suspected products. • % of licenced establishments and % of licenced personnel. • % of dispensing units inspected in a year.

Local health authorities can take several measures to promote the appropriate use of medicine, ensuring that medications are prescribed and used to maximize their health benefits. Local authorities can disseminate guidelines to healthcare providers and promote their integration into clinical practice.^79^ This needs to be combined with continuing education programmes and training sessions for health professionals on rational prescribing, medication safety and adherence-promoting strategies.^80^ Regular monitoring can help to gain insight into prescribing patterns, medication adherence rates and medication-related outcomes.^81,82^ In addition, local authorities need to collaborate with the organization that is responsible for enforcing regulations and policies related to pharmaceutical marketing, promotion and prescribing practices to prevent inappropriate use of medication.

The following indicators could be useful to assess appropriate use at the local level:

**Table:** 

• % of prescriptions that align with diagnosis and treatment guidelines. • % of patients receiving counselling on medication use, potential side effects and adherence. • % of patients who adhere to prescribed medication regimens. • Over-the-counter (OTC) sales of antibiotics and injections. • Assessment of patient-reported outcomes related to medication efficacy and use. • Educational interventions offered to healthcare providers reduce medication errors.

#### Monitoring system performance

A robust monitoring and evaluation system is essential for generating the data and insight needed for informed decision-making in LOPHAS. Adequate forecasting, planning, budgeting and other operations require reliant local information systems that are managed by adequately trained staff.^47^ The information gathered about pharmaceutical products and services at the local level provides valuable data for national health strategies and policies, but it is also crucial for local decision-making. Multiple studies indicate that by routinely analysing and utilizing data to inform decision-making and planning in pharmaceutical operations, local authorities can optimize resource allocation, improve operational efficiency and enhance the delivery of pharmaceutical services to communities.^24,55,63^

Reliable and timely publicly shared data are an important part of any attempt to improve transparency, accountability and responsiveness in pharmaceutical operations, including financial transactions and the effective use of resources.^5,83,84^ Ethical standards are important to ensure the integrity of professional behaviour. Clarifying and monitoring adherence to these standards is important for tackling corruption and improving efficiency, credibility and public trust in government institutions.

Indicators to measure this function need to assess the existence of monitoring systems that routinely measure data to identify problems and the use of these data to inform decision-making.

The following indicators could be useful to assess the local monitoring of system performance:

**Table:** 

• Local authorities routinely collect, analyse and share data about medicine availability, access to medicines and pharmaceutical services. • Local authorities indicate and oversee ethical standards for professional conduct and monitor compliance. • Local authorities routinely collect, analyse and share medicine revenue and expenditure data. • Local authorities routinely analyse and use data to inform decision-making and planning of pharmaceutical operations and policies.

#### Local factors influencing medicine access and availability

The local availability and accessibility of medicines are influenced not only by the organization of the pharmaceutical system but also by the local situation, including health needs and socioeconomic, cultural, logistical, geographical and security factors. A key feature of the local situation is the health-seeking behaviour of individuals, households and communities, influenced by their health status, preferences, economic situation and social, physical and cultural factors, all of which trigger demand for medicines and related services. Health status, needs and socioeconomic conditions, such as health insurance coverage, income levels and poverty rates, directly influence this demand.

Community members are not passive consumers but act as stewards of the system by demanding services and accountability and expressing their (dis)satisfaction with services and products. Historical experiences, cultural beliefs and practices also play a significant role; for example, preferences for traditional medicine, household spending priorities or mistrust of certain pharmaceuticals can impact which medicines are stocked and how they are used.^18,27^ Logistical factors, including the accessibility of health facilities, infrastructure quality, mark-ups and supply chain efficiency, influence medicines prices and the timely delivery and availability of medicines. In addition, the local security situation can significantly affect medicine availability, as conflict or instability can disrupt supply chains, limit access to health facilities and deter pharmaceutical companies from operating in certain areas.

When assessing and comparing the performance of LOPHAS, it is important to consider the influence of local circumstances. The purpose of the analysis and the specific context will determine which local factors to focus on, as these can vary significantly across different regions within a country.

Below, we list six key aspects of the local context that could be considered in an analysis of LOPHAS performance. For most aspects, relevant indicators and data are routinely available.

**Table:** 

• Health and demographic factors (e.g. population density, health needs, disease prevalence). • Socioeconomic conditions (e.g. health insurance coverage, income levels, employment). • Historical experiences, cultural beliefs and practices (e.g. traditional medicines practices, health beliefs and perceptions). • Transportation factors (e.g. distance to facilities, producers and distributors, transportation cost) • Geographical factors (e.g. physical barriers due to topography and terrain, weather conditions). • Security situation (e.g. security incidents, violence, law enforcement, political stability).

## Discussion

The aim of our study was to develop a systematic approach to analysing the performance of LOPHAS. We began developing the LOPHAS approach after observing significant regional variations in medicine availability within countries and recognizing that many key functions of pharmaceutical systems are co-managed or fully managed at the local level. The LOPHAS approach is designed to systematically learn from these local variations, generating insights to improve performance and access to medicines. This approach is particularly relevant for large, diverse countries with decentralized health systems.

Based on our integrative review and consultations with experts, we propose a four-step analytical approach supported by a framework of six core functions, each with operationalized components and indicators. By linking insights into the performance of local systems with key outcome metrics and contextual factors, we aim to facilitate learning through comparisons of local systems within a country, both cross-sectionally and over time. We expect this approach will help identify best practices and gaps and provide valuable insights that can be used to inform targeted actions for improving access to medicines.

In developing the LOPHAS approach, we drew upon various studies, frameworks and tools designed to measure various aspects of pharmaceutical systems. We noticed that the existing frameworks are grounded in three different types of system perspectives (mechanistic, teleological and CASs). These different types of system perspectives contain different ideas of what a system is and how systems emerge and evolve. We believe each perspective can offer valuable insights for analysing and strengthening LOPHAS.

The existing approaches and frameworks tend to focus on operations, policies and regulations at the national level. The LOPHAS approach specifically targets the subnational level, emphasizing the factors leading to healthcare access disparities within countries. While focusing on the national level, several frameworks recognize that operations, structures and dynamics at multiple levels are relevant to the performance of pharmaceutical systems.^14,15^ This is most evident in Bigdeli et al.’s^15^ approach, where the authors distinguish between stakeholders at five levels, including communities, service delivery stakeholders, the national policy and regulatory environment of the health sector and the national and international contexts. While we fully acknowledge the influence of national and international markets, regulations and dynamics, our focus is on the local level, where significant disparities exist that require targeted analysis and may offer opportunities for focused action.

Access to medicines is influenced not only by the functioning of the pharmaceutical system but also by local factors such as geographical barriers, logistical challenges, capacities and resources, poverty and insecurity. When analysing and comparing the performance of LOPHAS, it may be relevant to consider these local circumstances.

In two studies, where we tested elements of the broader LOPHAS approach, we analysed the influence of various local circumstances. In a study in Indonesia, we found that the availability of essential medicines was the lowest in primary health facilities in sparsely populated areas in the relatively poor districts in the east of the country, where the average distance to health facilities and medicine manufacturers was the greatest.^7^

A recent study using the LOPHAS approach in Afghanistan found that the local security situation had little impact on medicine availability.^42^ Instead, availability was significantly influenced by the type of organization managing the provincial health system. Provinces where health services were contracted to not-for-profit private organizations showed much higher availability of essential medicines compared to those managed directly by local governments. These findings underscore the value of analysing local differences to gain actionable insights that can inform the development of targeted policies and effective practices.

When applying the LOPHAS approach, analysts need to identify the most appropriate local level for their analysis. One way to do this is by mapping the subnational level at which key aspects of the pharmaceutical system are formally organized. This will likely depend on the structure of the health and pharmaceutical system. The relevant level may also differ depending on the type of medicine, as some products are distributed through national programmes, while others are locally sourced and provided. Another potential method for determining the most relevant local level and concerned local stakeholders is to analyse the geographical clustering of pharmaceutical outcome measures, such as medicine availability or affordability.

A key feature of the LOPHAS approach is the integration of quantitative analyses, which assess local variations in outcome measures (step 2), with in-depth qualitative studies that uncover actual practices in local pharmaceutical operations (step 3). This third step was introduced in response to empirical studies showing that diverse local dynamics and factors can unexpectedly affect the availability and accessibility of medicines, as well as the performance of LOPHAS.^27,40,85^ Where formal qualitative research capacities are limited in some local systems, strengthening these capacities or exploring alternative methods to gather and integrate qualitative feedback into the analysis can be beneficial.

An important trend is the rapid digitalization of pharmaceutical supply chains and healthcare facilities. When paired with efforts to harmonize systems, this digital transformation has the potential to significantly improve data availability for assessing the performance of LOPHAS.^75^ However, exclusive reliance on routine data may provide an incomplete or distorted picture of system functioning, as discrepancies often exist between documented procedures and real-world practices. To address this, integrating routine data with qualitative analyses is crucial for capturing the complexity of system dynamics, including the interplay of processes, incentives and practices. Such integrated understanding is essential for informing the design of interventions aimed at strengthening these systems.

The LOPHAS approach, framework and associated indicators are not intended as a fixed blueprint but as a rudimentary framework that needs to be operationalized and contextualized to make it useful. When adapting and applying the approach, the specific purpose of the system analysis, the needs of stakeholders and local circumstances need to be taken into account. As others have emphasized, a pharmaceutical systems framework is intended to be a tool that inspires, guides and focuses analyses. It should offer direction and help to structure thinking while allowing for creativity and adaptation based on the specific context or needs of the analysis

The LOPHAS approach appears particularly valuable for analysts, policymakers and other stakeholders in larger, more diverse countries that could benefit from a decentralized organization of pharmaceutical operations. For instance, our recent observations in Indonesia revealed that national regulations governing the financing and supply of medicines were effective in some regions but hindered access in others.^7^ These local variations underscore the need for a more decentralized, context-specific strategy to enhance access to medicines. The LOPHAS approach is expected to support the development of such strategies.

Systematic local data collection on medicine availability, as emphasized in the LOPHAS approach, is also vital for monitoring progress towards achieving SDG 3.8, which prioritizes equitable access to essential medicines.^86^ Currently, the SDG indicator 3.b.3, designed to measure access to medicines, is at risk of being discontinued due to insufficient data coverage.^87^ Enhancing local data collection efforts and integrating this information with broader health system performance metrics can help bridge these gaps, ensuring that progress towards this critical goal can still be effectively evaluated.

### Strengths and limitations

A strength of our study is that it builds upon an empirically grounded and comprehensive body of literature, combined with the rich experience of the consulted experts. We have included both grey and scientific literature based on studies in diverse countries that encompass a variety of perspectives. The included frameworks offer important conceptual insights into the components of pharmaceutical systems, while the empirical studies, reviews and expert consultations provide further evidence and experience with different empirical methodologies.

A limitation of this study is the lack of evidence specifically addressing LOPHAS within the broader context of national pharmaceutical and health systems. None of the included studies aimed to define a LOPHAS or assess the differences between the national and local dimensions. While local operations were mentioned, often indirectly, in several empirical studies, the limited focus on the local level highlights the importance of beginning each analysis with a comprehensive mapping of the local situation and applying the approach in a careful and reflective manner.

A second limitation results from the trade-off between complexity and pragmatism. Our analysis revealed numerous frameworks with extensive lists of stakeholders, components and overarching principles. We recognize that pharmaceutical systems are inherently complex and diverse and believe that it is not possible to encapsulate this complexity in a single framework. We have tried to develop an approach that incorporates the most relevant elements while being practically useful.

## Conclusion

In many countries, core functions of the pharmaceutical system are largely organized, delegated or co-managed at the local level. The LOPHAS approach and framework offer a structured tool for assessing and comparing these LOPHAS. Our four-step analytical approach recommends evaluating both the performance of LOPHAS and the contextual factors that influence access to medicines, combining quantitative analyses with in-depth qualitative studies of actual practices. Beyond its practical application, the LOPHAS framework holds scientific relevance as a means to systematically study LOPHAS – an area that remains understudied despite its growing importance, particularly in countries that have decentralized their health systems. We anticipate that such integrated studies and comparisons will yield valuable insights and inform concrete actions to improve access to medicines and, ultimately, advance universal health coverage.

## Supplemental Material

sj-docx-1-map-10.1177_27550834251371502 – Supplemental material for Assessing the performance of local pharmaceutical systems: An analytical approach to improve access to medicine Supplemental material, sj-docx-1-map-10.1177_27550834251371502 for Assessing the performance of local pharmaceutical systems: An analytical approach to improve access to medicine by Maarten Olivier Kok, Relmbuss Biljers Fanda, Rik Ubbo Lubbers, Margo van Gurp, Raffaella Ravinetto and Ari Probandari in The Journal of Medicine Access

sj-docx-2-map-10.1177_27550834251371502 – Supplemental material for Assessing the performance of local pharmaceutical systems: An analytical approach to improve access to medicine Supplemental material, sj-docx-2-map-10.1177_27550834251371502 for Assessing the performance of local pharmaceutical systems: An analytical approach to improve access to medicine by Maarten Olivier Kok, Relmbuss Biljers Fanda, Rik Ubbo Lubbers, Margo van Gurp, Raffaella Ravinetto and Ari Probandari in The Journal of Medicine Access
