# Supplementary material for: Assessing the performance of local pharmaceutical systems: An analytical approach to improve access to medicine
Source: J Med Access. 2025 Sep 27;9:27550834251371502. doi: 10.1177/27550834251371502 (PMC12476502; doi:10.1177/27550834251371502)
Supplement: sj-docx-1-map-10.1177_27550834251371502 – Supplemental material for Assessing the performance of local pharmaceutical systems: An analytical approach to improve access to medicine [file sj-docx-1-map-10.1177_27550834251371502.docx]

| **Appendix 1 – Analysis of framework domains*** | | | | | | | | | | | | | |
| --- | --- | --- | --- | --- | --- | --- | --- | --- | --- | --- | --- | --- | --- |
| Domains | Rapid pharmaceutical management assessment: an indicator-based approach (1995) (29) | | Improving ATM - a framework for collective action (2004) (30) | Functional components of a pharmaceutical system framework (2005) (31) | Using indicators to measure country pharmaceutical situations (2006) (13) | Regional framework on ATM in the Western Pacific (2009) (35) | Pharmaceutical supply management framework  (2012) (33) | ATM from a health system perspective: a conceptual framework (2013) (7) | SIAPS PSS framework (2013) (34) | Lancet commission progress indicators (2017) (35) | PSS measurement framework (2018) (36) | Action-oriented pharmaceutical sector strengthening cycle (2018) (32) | Access to medicines framework (2019) (11) |
| *Goals* |  | |  |  |  |  |  |  |  |  |  |  |  |
| - Access (accessibility, availability,   acceptability, affordability, quality) | X | | X | X | X | X | X | X | X | X | X | X | X |
| - Use (appropriate, rational) | X | | X | X | X | X | X |  | X | X | X | X | X |
| - Contribute to health outcomes/status |  | |  | X |  |  |  | X | X |  | X |  |  |
| - Coverage | X | |  | X |  |  |  |  | X |  | X |  | X |
| - Efficiency | X | |  | X |  |  |  |  |  |  | X |  |  |
| - Social and Financial protection |  | |  | X |  |  |  |  |  |  |  |  | X |
| - Responsiveness |  | |  |  |  |  |  |  |  |  | X |  |  |
| - Satisfaction |  | |  | X |  |  |  |  |  |  | X |  |  |
| *Products accessed and used* | |  |  |  |  |  |  |  |  |  |  |  |  |
| - Medicines | X | | X | X | X | X | X | X | X | X | X | X | X |
| - Pharmaceutical products | X | |  | X | X |  | X |  | X |  | X | X | X |
| - Medical products |  | |  |  | X |  | X |  |  |  |  |  |  |
| - Vaccines |  | |  |  |  |  |  |  |  |  |  |  | X |
| - Health Technologies |  | |  |  |  |  |  |  |  |  |  |  |  |
| - Pharmaceutical services | X | |  | X | X | X |  |  | X |  | X | X | X |
| *Characteristics of products accessed and used* | | | | |  |  |  |  |  |  |  |  |  |
| - Quality | X | | X | X | X | X | X | X | X | X | X | X | X |
| - Essential |  | | X |  | X | X |  |  |  | X |  |  | X |
| - Safe/safety | X | |  | X | X |  | X |  | X | X | X | X | X |
| - Effective (efficacy) | X | |  |  | X |  | X |  | X |  | X | X |  |
| - Cost-effective |  | |  |  |  |  | X |  |  |  | X |  |  |
| *Qualities associated with access and use* | | |  |  |  |  |  |  |  |  |  |  |  |
| - Quality | X | |  | X | X | X | X | X | X | X | X | X | X |
| - Safe/safety | X | |  | X | X | X |  |  |  | X |  | X | X |
| - Effective (efficacy) |  | |  |  | X |  |  |  | X |  | X | X |  |
| - Cost-effective |  | |  |  |  |  |  |  |  |  |  |  |  |
| *Overarching principles* |  | |  |  |  |  |  |  |  |  |  |  |  |
| - Equity |  | |  | X | X | X |  | X | X | X | X | X |  |
| - Timeliness |  | |  | X |  |  |  |  |  |  |  |  | X |
| - Human rights |  | |  |  |  |  |  | X |  | X |  | X |  |
| *Stakeholders* |  | |  |  |  |  |  |  |  |  |  |  |  |
| - Structures/institutions/   Organizations |  | |  |  |  |  |  |  |  |  |  |  |  |
| - Individuals/people |  | |  |  |  |  |  | X |  |  |  | X |  |
| - Government | X | | X | X | X | X |  |  | X | X | X | X | X |
| - Providers | X | | X | X | X | X |  |  | X | X | X | X | X |
| - Communities and households | X | |  | X | X | X |  | X | X | X | X | X | X |
| - Public sector |  | | X | X | X |  |  |  |  |  |  |  |  |
| - Private sector | X | | X | X | X |  |  |  |  |  |  |  |  |
| - International, national, subnational, and local |  | |  |  |  | X |  | X |  | X |  |  |  |
| *Functions (subsystems)* | | |  |  |  |  |  |  |  |  |  |  |  |
| - Selection |  | |  |  |  |  | X |  |  |  |  |  |  |
| - Procurement |  | |  |  |  |  | X |  |  |  |  |  |  |
| - Distribution |  | |  |  |  |  | X |  |  |  |  |  |  |
| - Use |  | |  |  |  |  | X |  |  |  |  |  |  |
| - R&D |  | |  |  |  |  |  |  |  |  |  |  |  |
| - Regulation |  | |  |  |  |  |  |  |  |  |  |  |  |
| - Manufacturing |  | |  |  |  |  |  |  |  |  |  |  |  |
| *System components* | | |  |  |  |  |  |  |  |  |  |  |  |
| - Service delivery | X | | X | X | X | X |  | X | X |  | X | X | X |
| - Leadership and governance |  | |  | X | X | X | X | X | X |  | X |  |  |
| - Policies, law, and regulation | X | | X | X | X | X | X |  |  |  | X | X |  |
| - Resources:   - Medical products, vaccines, health technologies | X | |  |  | X | X |  | X | X |  | X |  |  |
| - - Human/resources, health workforce | X | |  | X | X | X | X | X | X |  | X | X | X |
| - - Information | X | |  |  | X |  | X | X | X |  | X | X | X |
| - - Financing (price setting/negotiation) | X | | X | X | X | X | X | X | X |  | X | X | X |
| - - Infrastructure |  | |  |  |  |  |  | X |  |  |  |  |  |
| - - Organization | X | |  |  |  |  | X |  |  |  |  |  |  |
| *Context* |  | |  |  |  |  |  |  |  |  |  |  |  |
| - Market forces |  | |  |  |  |  |  | X |  |  |  |  |  |
| - Innovation |  | |  |  |  |  |  | X |  |  |  |  |  |
| - Transparency |  | |  |  |  |  |  | X |  | X |  |  |  |
| - Donor agenda/funding |  | |  |  |  |  |  | X |  | X |  |  |  |
| *Table structured similar to Hafner et al. (6) | | | | | | | | | | | | | |
