# Supplementary material for: Assessing the performance of local pharmaceutical systems: An analytical approach to improve access to medicine
Source: J Med Access. 2025 Sep 27;9:27550834251371502. doi: 10.1177/27550834251371502 (PMC12476502; doi:10.1177/27550834251371502)
Supplement: sj-docx-2-map-10.1177_27550834251371502 – Supplemental material for Assessing the performance of local pharmaceutical systems: An analytical approach to improve access to medicine [file sj-docx-2-map-10.1177_27550834251371502.docx]

Supplementary file 2

| **Empirical studies explicitly using a Pharmaceutical Systems perspective** | | | |
| --- | --- | --- | --- |
| **Authors** | **Aim(s)** | **Study type** | **Quality appraisal** |
| Zagorski et al. (27) | Identify areas of irrational drug management in the pharmaceutical supply system with respect to selection, procurement, distribution and use of drugs, including aspects of finance, policy, and law, as they affect drug management. | Assessment | High |
| Emmerick et al. (91) | The objective of this study is to explore and analyse the results of the Diagnosis of the Pharmaceutical Situation in Brazil (DiagAF-Br) from a regional perspective for access to medicines, use and quality. | Assessment | High |
| Wiedenmayer (92) | To describe the current pharmaceutical situation in Tanzania and Mozambique using the 4 components from the WHO’s framework for equitable access to medicine; selection, affordable prices, sustainable financing and reliable health and supply systems. | Assessment | N/A report |
| Gray et al. (62) | Describe the background and current position of pharmacy in South Africa using the WHO’s building block framework. | Assessment | N/A |
| Ooms et al. (28) | To assess barriers in accessing internationally controlled essential medicines in Uganda by conducting semi-structured interviews with key stakeholders using the framework by Bigdeli et al. (16) to analyse the results | Barriers & enablers | High |
| Zaidi et al. (30) | To analyse barriers in accessing essential medicines in Pakistan using the WHO’s framework for equitable access to essential medicine (86) | Barriers & enablers | High |
| Seoane-Vazquez (69) | To describe the current pharmaceutical situation in Guyana and identify barriers in access to medicines using the WHO’s framework for equitable access to essential medicine (86) | Barriers & enablers | Medium |
| Bhuvan et al. (26) | To conduct a health facility based study on access to medicines combined with semi-structured interviews using the WHO’s operational package for assessing, monitoring and evaluating the countries pharmaceutical situation (25) | Barriers & enablers | High |
| Bigdeli et al. (66) | To determine health system barriers to the access and use of Magnesium Sulphate for women with severe pre-eclampsia and eclampsia in Pakistan using a fishbone diagram | Barriers & enablers | High |
| Barillas (87) | To analyse two interventions in Ecuador and Guatemala in a pharmaceutical system using a system approach, providing also their own framework | Intervention | N/A report |
| Luiza et al. (29) | To analyse the impact of the Farmácia Popular Program (FPP) in Brazil using a health system perspective using the framework by Bigdeli et al. (16) | Intervention | High |
| Oteba et al. (61) | To assess systemwide interventions to strengthen Uganda’s pharmaceutical system conducted under the acronym: Securing Ugandans Rights to Essential Medicines (SURE) using their own framework | Intervention | N/A |
| Prashanth et al. (93) | To understand health system factors for improving equitable access to quality generic medicines using the framework by Bigdeli et al. (16) | Intervention | N/A |
